# Evaluation of the Relevance of Piaget’s Cognitive Principles among Parented and Orphan Children in Belagavi City, Karnataka, India: A Comparative Study

**DOI:** 10.5005/jp-journals-10005-1463

**Published:** 2017-02-27

**Authors:** Chandrashekhar M Badakar, Prachi J Thakkar, Shivayogi M Hugar, Pratibha Kukreja, Harsha G Assudani, Niraj Gokhale

**Affiliations:** 1 Reader, Department of Pedodontics & Preventive Dentistry, KLE VK Institute of Dental Sciences, KLE University, Belagavi, Karnataka India; 2 Postgraduate Student, Department of Pedodontics & Preventive Dentistry, KLE VK Institute of Dental Sciences, KLE University, Belagavi, Karnataka India; 3 Professor, Department of Pedodontics & Preventive Dentistry, KLE VK Institute of Dental Sciences, KLE University, Belagavi, Karnataka India; 4 Postgraduate Student, Department of Pedodontics & Preventive Dentistry, KLE VK Institute of Dental Sciences, KLE University, Belagavi, Karnataka India; 5 Postgraduate Student, Department of Pedodontics & Preventive Dentistry, KLE VK Institute of Dental Sciences, KLE University, Belagavi, Karnataka India; 6 Lecturer, Department of Pedodontics & Preventive Dentistry, KLE VK Institute of Dental Sciences, KLE University, Belagavi, Karnataka India

**Keywords:** Centration, Child, Cognitive, Comparative, Ego-centrism, Parent.

## Abstract

**Aim:**

To determine and compare the relevance of Piaget’s cognitive principles among 4- to 7-year-old parented and orphan children in Belagavi City, Karnataka, India.

**Materials and methods:**

This study was conducted on 240 children between the ages of 4 to 7 years who were equally divided into two groups of 120 parented and 120 orphan children. These were subdivided into four groups of 30 children each. Various characteristics like egocentrism, concept of cardinal numbers based on centration, lack of conservation, and reversibility were assessed, using experiments and comparison of their prevalence between two groups was carried out.

**Results:**

There is a statistically significant difference in the cognitive development among parented and orphan children age 4 to 7 years.

**Conclusion:**

There is a significantly better cognitive development among parented children as compared with orphan children in Belagavi city.

**Clinical significance:**

A child is not a miniature adult but rather can think and perceive the world differently from an adult. Understanding a child’s intellectual level can enable a pedodontist to deliver improved quality care to children. According to Jean Piaget, in the preoperational period, children think symbolically and their reasoning is based more on appearance rather than logic. It is often rightly said that a child’s behavior is a reflection of his parents. However, Piaget did not consider the effect of social setting and culture on the cognitive development. This study was carried out as there is not much literature available to describe the cognitive development of children in the Indian scenario and the influence of parental presence on the same.

**How to cite this article:** Badakar CM, Thakkar PJ, Hugar SM, Kukreja P, Assudani HG, Gokhale N. Evaluation of the Relevance of Piaget’s Cognitive Principles among Parented and Orphan Children in Belagavi City, Karnataka, India: A Comparative Study. Int J Clin Pediatr Dent 2017;10(4):346-350.

## INTRODUCTION

There are some subtle, and yet important aspects of dental practice which help to guide children through their dental experiences successfully. It is generally agreed that if the dentist is to perform satisfactory dental care for child patients, the dentist must have their full cooperation and establish positive and non-threatening relationships. Chambers suggests that communication is the key to establishing this rapport with patients.^1^ Thus, to communicate successfully with a child, it is necessary to understand his or her intellectual level and the way in which thought processes work. As per Jean Piaget’s theory of cognitive development, the development of intellectual capabilities occur in a series of relatively distinct stages. A child’s way of thinking about and viewing the world is quite different at the different stages.^2^ In the second stage of cognitive development which is the preoperational period, children think symbolically about objects, and reason is based more on appearance rather than logic.^3^

Research on the cognitive development of the child and its application to dental health service can enable us as Pediatric dentists to better understand them and deliver improved quality of care.^4^

There is a debate within psychology on “Nature v/s Nurture” which is concerned with the extent to which inherited (i.e., genetic) or acquired (i.e., learned) characteristics contribute to particular aspects of behavior.^5^

Attachment allows children the “secure base” necessary to explore, learn, and the motivation and opportunity to do so. Children’s attachment patterns are highly influenced by those of their parents. The attachments of both child and parents affect children’s physical, developmental, psychological, and behavioral wellbeing.^6^

In the cognitive theory that was put forth in 1952, Jean Piaget emphasized on the universal stages of cognitive development and biological maturation; however, he has not considered the effect that the culture and social setting may have on the cognitive development.^7^

Thus the aim of this study was to determine whether the social environment of the child has any influence on their cognitive development and compare the relevance of Piaget’s cognitive principles among 4- to 7-year-old parented and orphan children in Belagavi city.

## MATERIALS AND METHODS

The study was conducted after obtaining prior approval from the Institutional Review Board and Ethical Committee. One part of this study was conducted in a school in Belagavi, prior to which, permission was obtained from the principal of the school and a letter of consent for the child’s participation was obtained from the parents.

The second part of the study was conducted in the Orphanage for which permission was obtained from the concerned authority of the orphanage.

### Inclusion Criteria

- 4- to 7-year-old healthy children.

### Exclusion Criteria

- Specially abled children.
- Children whose parents refused to give consent for participation in the study.

Since no study was previously done to compare the cognitive development among parented children and orphan children, as per convenience sampling, this study was conducted on 120 orphan children in Belagavi city in the age group of 4 to 7 years (all samples available in the city in that age group) and compared with 120 parented children of age 4 to 7 years.

*Group I:* 120 parented children (an attempt was made to include children of similar socioeconomic status from the same school)

*Group II:* 120 orphan children

The 120 children from each group were further divided into four groups of 30 children each based on their age:

1. 4 years: 30 children
2. 5 years: 30 children
3. 6 years: 30 children
4. 7 years: 30 children

Three experiments were conducted to assess the classical characteristics of children belonging to this age group:

1. Egocentrism,
2. Lack of concept of cardinal numbers based on centra-tion, and
3. Lack of conservation and reversibility.

**Fig. 1:**
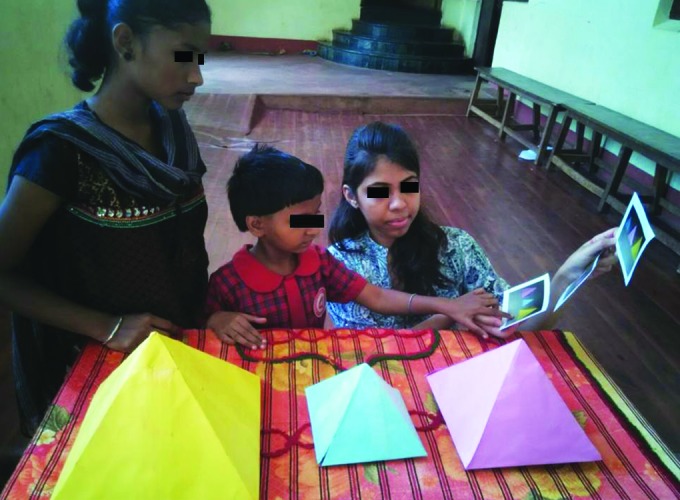
Classical three mountain experiment

### Egocentrism

It is the inability to take the perspective or point of view of another person.

Assessment of this was done by the classical three mountain experiment as described by Piaget. The child was made to sit on one side of the table with a doll placed on the other side of the table on which three mountains of different heights were placed. As shown in Figure 1, the child was asked to select one photograph from a series, which reflected the doll’s view.

If a child picked a picture reflecting their own view rather than the doll’s view he/she was unable to understand another person’s point of view and was thus egocentric.^4^

### Concept of Cardinal Numbers based on Centration Principle

Centration is the tendency to focus, or centre, only on one aspect of a situation and other aspects of the situation are ignored.

**Fig. 2:**
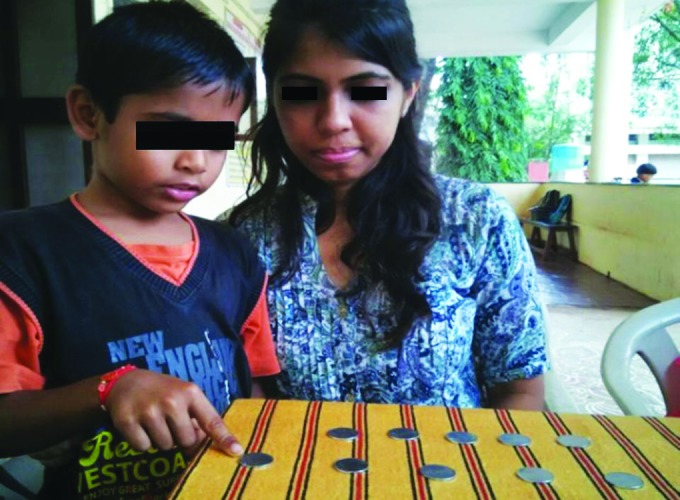
Child points out to the row of spread out coins and shows it has more number of coins

The coin experiment was used to assess the concept of cardinal numbers. The child was shown two rows of same number of coins and asked if both the rows had equal number of coins. The second row of coins was then spread out a little and the child was asked the same question as above (Fig. 2). The child with the concept of centration pointed out that the spread out row had more number of coins, whereas the child with the concept of cardinal numbers answered that both rows had equal number of coins.^4^

### Lack of Conservation and Reversibility

The principle of conservation explains that as long as nothing is added or subtracted two equal physical quantities remain equal even if the appearance of one is changed.

**Figs 3A and B:**
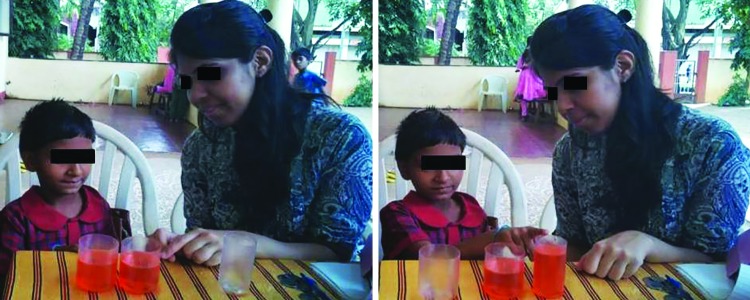
Child chooses a taller and narrower beaker as to having more amount of colored liquid

The concept of conservation was assessed by the beaker experiment. The child was presented with the same amount of colored liquid in two identical beakers. Then the liquid was poured from one beaker into a third thinner and taller beaker. Then the child was asked to identify the beaker that contained more amount of liquid (Fig. 3). If the child pointed out the taller beaker as the one containing more liquid, he/she was considered to lack the concept of conservation.^4^

The data thus obtained were statistically analyzed by the SPSS 18 software using frequency distribution and chi square test.

## RESULTS

Table 1 shows the prevalence of three characteristics in both the groups. There is a statistically significant difference in the prevalence of egocentrism and lack of concept of cardinal numbers among the parented and orphan children. However, the difference in the lack of conservation is not statistically significant among the two groups.

**Graph 1:**
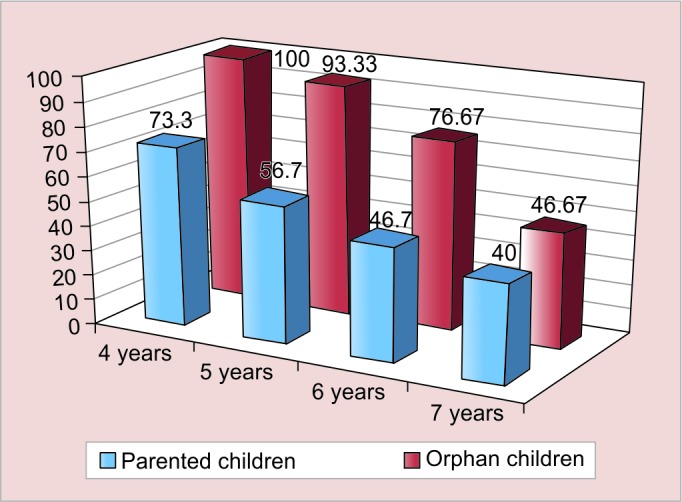
Prevalence of egocentrism based on the three mountain experiment among different ages in both the groups

**Graph 2:**
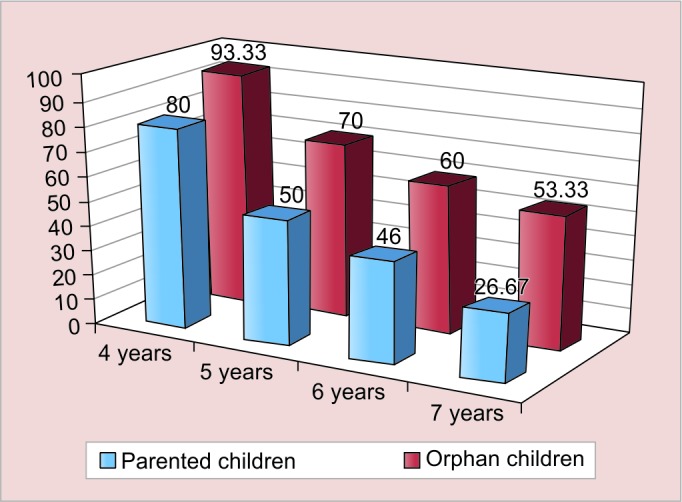
Prevalence of lack of concept of cardinal numbers based on the coin experiment among different ages in both the groups

**Graph 3:**
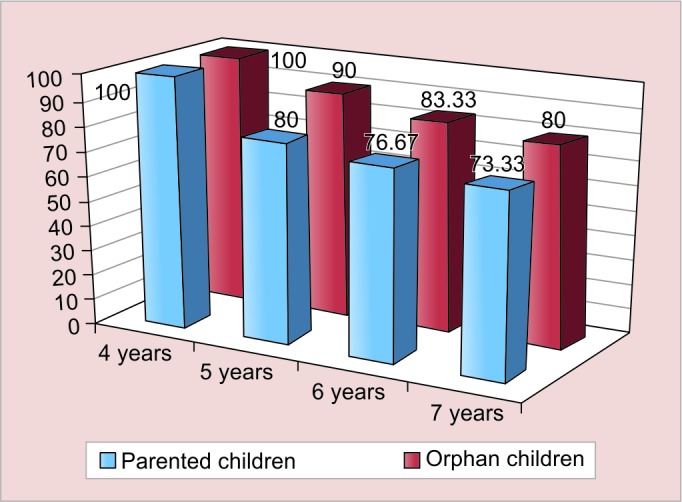
Prevalence of lack of conservation based on the beaker experiment among different ages in both the groups

Graphs 1 to 3 show the agewise comparison of the three characters of both the groups.

Each of the three characters shows a gradual reduction in prevalence with increase in age. There is a statistically significant difference in the egocentrism of parented and orphan children in the age group of 4 years (p = 0.002), 5 years (p = 0.001), and 6 years (p = 0.017).

However, the differences in the lack of concept of cardinal numbers and lack of conservation are not statistically significant in the individual age groups.

**Table 1:** **Table 1:** Prevalence of the three characteristics tested in both the groups

		*Prevalence among parented children (total 120)*		*Prevalence among orphan children (total 120)*	
Egocentrism*		65		95	
Lack of concept of		59		83	
cardinal numbers*					
Lack of conservation		98		106	
and reversibility					

*Statistically significant difference among the two groups

## DISCUSSION

Prelogical reasoning appears in the intuitive stage of the preoperational period as described by Jean Piaget in the cognitive theory of development. The child begins to construct more elaborate concepts and more complex images.^4^ These intuitive stage children pose a challenge to the dentists as they could be a behavior management problem and were thus included in the study.

In a study conducted by Asokan et al^4^ to assess the relevance of Piaget’s cognitive principles among children of 4 to 7 years, it was observed that the characteristic features explained by Piaget were present in most of the children between ages of 4 and 7 years.

Vygotsky^7^ places more emphasis on culture and social factors having effect on cognitive development. The environment in which children grow up has an influence on how and what they think about. Adults become an important source of cognitive development wherein the culture’s tools of intellectual adaptation that they transmit are internalized by children. Thus social interaction with a skilful tutor contributes to much important learning by the child.^8^

Freund conducted a study which supported this concept in which he found that those children who had previously worked with their mother showed greater improvement compared with their first attempt at the task alone. Thus the conclusion was that guided learning within the zone of proximal development led to greater understanding/performance than working alone.^9^

The learning of a child begins long before he/she enters school. The human learning presupposes a specific social nature and a process by which children grow into the intellectual life of those around them.^7^ Also a study has shown that linguistic differences may affect cognitive development.^10^

As shown in Graph 1, there is as statistically significant difference in the egocentrism and concept of cardinal numbers among the parented and orphan children of 4 to 7 years age.

The notion of an egocentric mode of spatial representation in the young child was first described by Piaget and Inhelder.^11^ Inability to represent the view seen by another observer in a different orientation has generally been seen in the developmental psychology literature as a failure in social cognition.

In a dental setting, it would not be of use to point out to a child that, his parents would be proud of him, if he stopped the digit sucking habit because the child thinks that his parent’s attitude about digit sucking would be exactly the same as his own.^2^ Taking advantage of his egocentrism, the child could be allowed to make believe he/she important and could be permitted to take some decisions regarding the treatment, e.g., when to stop temporarily using hand signals or allow them to be “in-charge” of the saliva ejector.^12^

Concept of cardinal numbers, conservation, and reversibility are based on the concept of centration. Children were misled by the most striking feature, the length of the row and the height of the beaker. The child could not simultaneously consider the height and the width of the liquid in the container due to centration and the child between ages 2 and 7 cannot decenter.^12^ They have difficulty in solving problems because they cannot consider all the aspects involved in the problem. Thus, during dental treatment, the child should be directed to focus on the hand mirror given to him and concentrate in watching the procedure. This will tend to distract him/ her from the horrifying instruments.^13^

Distraction is the key for practicing painless dentistry. Audio and visual distractions like head phones with pleasant music, the presence of an aquarium next to the dental chair, could reduce dental anxiety, make them relaxed and divert the child’s attention from the noise of the drill.^14^ The child at this stage of development generally focuses on the most striking and exciting features in the dental setup.

## CONCLUSION

Through this comparative study we can conclude that parental presence or absence has a significant impact on the cognitive development of the child. In this study it is observed that a significantly greater number of orphan children show egocentrism and lack the concept of cardinal numbers than parented children in the age group of 4 to 7 years. Also there is a gradual and uniform reduction in the prevalence of the three characters studied with increase in age from 4 to 7 years.

## CLINICAL SIGNIFICANCE

Hence, providing the children a colorful, relaxing, and friendly environment to focus on distracts them from the apparently “terrifying” instruments present in a dental setting.^4^

This study has shown the influence of parental presence/absence on cognitive development of children. However, further research is warranted to study the influence of other factors on cognitive development. We also need to consider that orphan children need to be paid more individual attention and be provided with better teaching facilities and exposure to recent/newer modes of learning.
